# A Co-Expressed Cluster of Genes in the Anterior Brain of Female Crickets Activated by a Species-Specific Calling Song

**DOI:** 10.3390/ijms27020706

**Published:** 2026-01-10

**Authors:** Shijiao Xiong, Chunxia Gan, Fengmin Wang, Zhengyang Li, Songwang Yi, Yaobin Lu, Xinyang Zhang

**Affiliations:** Xianghu Laboratory, Hangzhou 311231, China; xiongshijiao@zju.edu.cn (S.X.);

**Keywords:** cricket, auditory processing, pulse pattern recognition, transcriptome analysis, gene co-expression network

## Abstract

Crickets use the pulse pattern of the species-specific calling song as a primary cue for mate recognition. Here we combined transcriptome profiling of brain regions with network-based analyses in *Gryllus bimaculatus* exposed to silence or pulse trains known to elicit strong or weak phonotactic attraction. Acoustic stimulation triggered specific transcriptional changes in the brain, with the anterior protocerebrum showing the most pronounced and selective responses to the calling song pattern, characterized by enrichment in neuromodulatory and neurotransmitter-related pathways. Weighted gene co-expression analysis identified a specific cluster of highly co-expressed genes in the anterior brain (termed the calling song-responsive module) that responded selectively only to the calling song stimulus. Genetic network topology analysis revealed six highly connected key hub genes within the calling song-responsive module—*GbOrb2*, *Gbgl*, *Gbpum*, *GbDnm*, *GbCadN*, and *GbNCadN*. These genes showed extensive interactions with many other genes in the network, suggesting their central regulatory role in response to calling song in female crickets. These findings support the anterior brain as a central integrator of cricket auditory mate recognition cues and point to a core molecular network that likely underpins this behavior.

## 1. Introduction

Acoustic communication is crucial for the survival and reproduction of many animals, facilitating species recognition, mate localization, and predator avoidance [1]. The field cricket, *Gryllus bimaculatus*, has long been used as a neuroethological model due to their distinct phonotactic behavior for dissecting the underlying neural and molecular mechanisms [2,3]. Male field crickets produce a species-specific calling song with a distinct temporal pattern consisting of chirps with 4 ± 1 pulses, repeated at a rate of 2–3 Hz [4]. Female crickets recognize this conspecific temporal pattern and perform positive phonotaxis to locate the singing male [5,6]. This communication system depends on the fine-tuning between the acoustic features of the calling song, and the female’s auditory system [7,8].

Behavioral assays show that species-specific pulse periods (34–42 ms) elicit maximal phonotactic responses (>90% of maximum response strength), whereas sound patterns shifted away from the conspecific pattern (both shorter 10–18 ms and longer 58–90 ms pulse periods) only produce minimal responses (<20% of maximum response strength) [5]. Neurophysiological work has traced the auditory processing from the prothoracic ganglion to the protocerebrum, where specialized neural circuits mediate sound pattern recognition and drive selective phonotactic behavior in females [5,9,10].

Despite this detailed understanding of the neural basis and behavioral output, the underlying molecular mechanisms controlling the behavior remain largely unknown. While transcriptomic analyses have provided valuable insights into auditory processing in other insects like *Drosophila* [11] and locusts [12], similar studies in crickets are still lacking, leaving the molecular basis of phonotactic behavior unexplored. The recent availability of *G*. *bimaculatus* genome, however, allows a systematic investigation of how gene expression in response to acoustic stimuli correlates with behavioral decision-making [13].

Here, we aim to characterize transcriptional changes in the cricket brain that are linked to processing of the conspecific calling song. We profiled transcriptomic responses of two different brain regions to four distinct acoustic stimuli: a no-sound control; two behaviorally unattractive songs with abnormally short (10 ms) or long (80 ms) pulse periods, both of which elicit minimal phonotactic responses; and the species-specific calling song (40 ms pulse period) that elicits the strongest response. Using high-throughput RNA sequencing, we characterized the transcriptional responses across these acoustic conditions, revealing both region-specific patterns and stimulus-dependent gene expression changes. By comparing gene expression patterns between behaviorally attractive and unattractive acoustic stimuli, this study aims to identify candidate genes and molecular pathways that may contribute to the transcriptional regulation of auditory pattern recognition.

## 2. Results

### 2.1. Female Crickets Preferably Respond to Calling Song Stimulus

**Figure 1:**
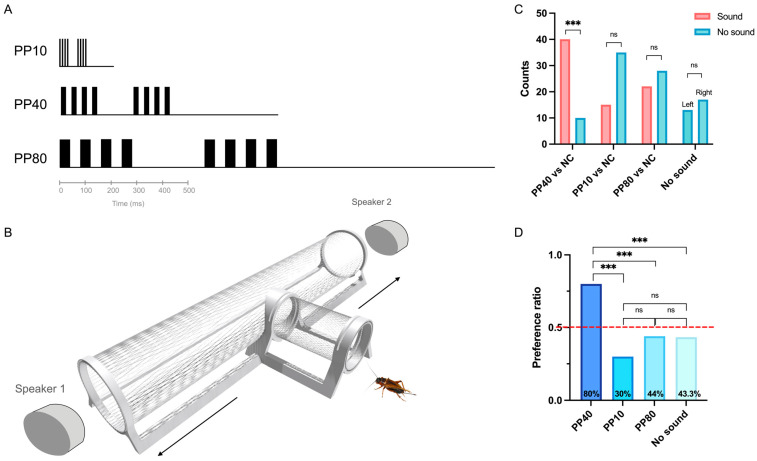
T-maze choice behavioral assay for to acoustic stimulus responses in *Gryllus bimaculatus*. (**A**) Schematic representation of acoustic stimuli used in the study. Three synthetic pulse patterns were tested: PP10 (10 ms pulse period), PP40 (40 ms pulse period, species-specific calling song analogue), and PP80 (80 ms pulse period). Each chirp consisted of four sound pulses (colored rectangles) separated by silent intervals. For all patterns, pulse duration and interval duration were equal (50% duty cycle). Two consecutive chirps are shown to illustrate the repetitive structure of the stimulus. Inter-chirp intervals were equal to one chirp period for each pattern. (**B**) Schematic of the T-maze behavioral choice assay used to assess cricket preference for different pulse patterns. (**C**) Behavioral choice results showing the number of females selecting either the acoustic stimulus (red bars) or the control (blue bars). Bars represent counts of individual females choosing each option. Four contrasts were tested: PP40 vs. NC, PP10 vs. NC, and PP80 vs. NC, no sound control. Within-group comparison of attracted versus not attracted trials across four experimental conditions. Asterisks indicate significance levels from binomial tests: *** *p* < 0.001 (Bonferroni corrected), ns = not significant. (**D**) Choice proportions across experimental conditions. The dashed red line indicates 50% chance level (random choice). Horizontal brackets with asterisks indicate significant pairwise differences (Bonferroni corrected): *** *p* < 0.001. Only the PP40 condition showed a choice proportion significantly above chance level (χ^2^ = 27.25, df = 3, *p* < 0.001).

Crickets robustly discriminated between temporal pulse patterns, showing a significantly stronger phonotactic responses to the calling song-like stimulus (PP40; Figure 1A) than weaker alternatives (PP10, PP80; Figure 1A) or no sound controls. In choice trials (Figure 1B), 40/50 females chose PP40 (*p* < 0.001, binomial test), demonstrating strong preference for this pattern. In contrast, only 15/50 females chose PP10 and 22/50 chose PP80, neither of which significantly differed from their respective no-sound controls (Figure 1C). Crickets showed no directional preference in the no-sound control trials. Consistent results were obtained when choices were expressed as proportions (Figure 1D). A chi-square test revealed significant differences in choice proportions among the four conditions (χ^2^ = 27.25, df = 3, *p* < 0.001). Post hoc pairwise comparisons with Bonferroni correction showed that the PP40 condition elicited significantly higher attraction responses (80%) compared to all other conditions (all *p* < 0.01). The high response rate to PP40 indicates that nearly all tested individuals were capable of detecting and responding to the calling song pattern, ensuring that subsequent transcriptional analyses of individuals from the same colony captured brain states associated with active auditory processing rather than non-responsiveness.

These behavioral data demonstrate that the specimen used for the molecular analysis very likely showed a clear behavioral preference for PP40, whereas the other pulse patterns elicited only a weak or no consistent responses. This allowed us to investigate molecular responses of the brain associated with calling song processing.

### 2.2. Anterior Brain Tissue Exhibits Strongest Transcriptional Response to Attractive Sound

**Figure 2:**
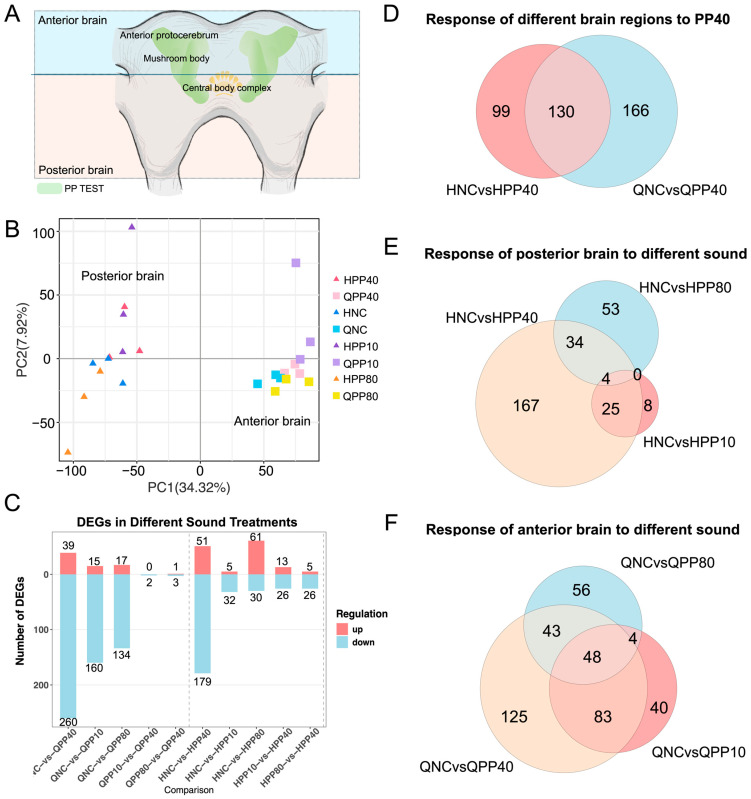
Differentially expressed genes in response to different acoustic stimuli across tissues. (**A**) Anatomical diagram showing the dissection of cricket brain tissue with a horizontal cut delineating the anterior brain (Q) and posterior brain (H) regions for RNA sequencing analysis. (**B**) Principal component analysis (PCA) of RNA-seq samples from anterior brain (Q), and posterior brain (H) tissues under four acoustic conditions: NC (no sound control), PP40, PP10, and PP80. (**C**) Numbers of upregulated and downregulated differentially expressed genes (DEGs) in each tissue type in response to different acoustic stimuli. (**D**) Venn diagram illustrating the overlap of DEGs across posterior brain, and anterior brain tissues in response to PP40. (**E**) Venn diagram showing the overlap of DEGs in posterior brain tissue (H) in response to PP40, PP10, and PP80 stimuli. (**F**) Venn diagram displaying the overlap of DEGs in anterior brain tissue (Q) across the three acoustic stimuli.

To identify the transcriptional correlates of selective auditory behavior, we profiled RNA-seq data from the anterior and posterior parts of the brain under four acoustic conditions (Figure 2A). Principal component analysis (PCA) revealed clear sample clustering by brain region type, with PC1 separating the anterior brain from the posterior brain (Figure 2B, 34.32% variance explained). Within each tissue sample, different acoustic stimuli induced activation of additional subclusters, indicating stimulus-specific transcriptional responses.

Analysis of differential expressed genes showed that the anterior part of the brain exhibited the most extensive response to the PP40, with 299 differentially expressed genes (39 upregulated and 260 downregulated). In comparison, the posterior part of the brain responded with 230 differentially expressed genes (DEGs; 51 up/179 down; Figure 2C).

Venn diagram analyses further highlighted the anterior brain’s particular response to PP40, with 269 DEGs identified compared to 229 in the posterior brain (Figure 2D). Among these, 130 DEGs were shared across brain regions, while 166 and 99 were unique to the anterior and posterior brain, respectively. Within the posterior brain, different acoustic stimuli elicited largely non-overlapping DEG sets (Figure 2E). By contrast, the anterior brain maintained a core set of 48 DEGs were shared across all 3 tested pulse patterns (Figure 2F), suggesting a stable transcriptional module for supporting species-specific general auditory signal processing.

KEGG and GO enrichment analyses point to region-specific molecular responses to PP40. In the anterior brain, PP40 stimulation was associated with enrichment of metabolic and immune-related pathways. KEGG pathway analysis identified drug metabolism and biosynthesis of secondary metabolites, with UDP-glucuronosyltransferase (UGT) and carboxylesterase families significantly enriched (Figure S1A). GO enrichment analysis revealed immune defense–related terms, including innate immune response and immune response-activating signal transduction (Figure S1B). These results suggest that molecular responses in the anterior brain to attractive sound involve an interplay between immune activation and regulation of endogenous compounds. In the posterior brain, PP40 was associated with enrichment of metabolic processes. KEGG pathways included tyrosine metabolism, betalain biosynthesis, and isoquinoline alkaloid biosynthesis, with tyrosinase (TYR) genes as key contributors (Figure S1A). GO enrichment showed terms such as carboxylic acid metabolic process and glucose transport (Figure S1B).

These results demonstrate that PP40 activates coordinated yet brain region-specific molecular changes, with the anterior brain showing enrichment in immune and metabolic pathways, and the posterior brain in metabolic processes.

### 2.3. Identification of a Sound-Induced Co-Expression Module in the Anterior Brain

**Figure 3:**
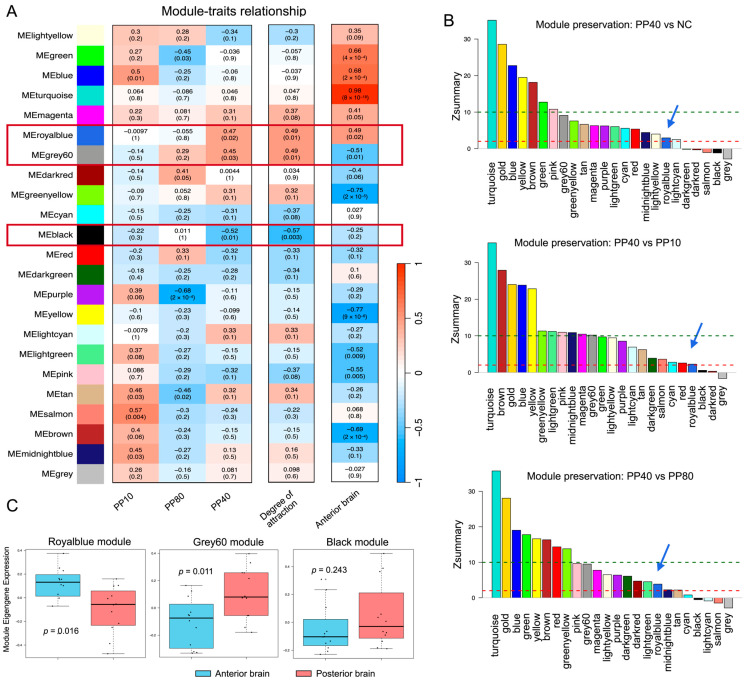
Identification and co-expression similarity of sound-responsive modules in the anterior brain. (**A**) Module-trait relationships between WGCNA-identified co-expression modules and five traits categorized into three groups: acoustic stimuli (PP10, PP80, PP40), behavioral degree of attraction, and brain region (anterior brain). The colored modules group together genes with highly correlated expression profiles, where each color represents a specific module of co-expressed genes. The grey module consists of all genes not classified into any other module. Rows represent gene modules and columns represent traits. Color intensity indicates the strength of Pearson correlation between module eigengenes and traits (red, positive correlation; blue, negative correlation). Numbers in each cell display correlation coefficients and their corresponding *p*-values. Red boxes highlight modules that show significant correlations with PP40 and/or behavioral preference. (**B**) Module preservation analysis across acoustic conditions. Module preservation statistics were calculated using the modulePreservation() function in WGCNA, comparing PP40 with NC, PP10, and PP80. The bar plot displays the Zsummary score for each module, with colors corresponding to module identities. Zsummary < 2 indicates non-preserved modules, 2–10 indicates low preservation, and >10 represents high preservation. Red and green dashed lines parallel to the *X*-axis indicate the thresholds of Zsummary = 2 and Zsummary = 10, respectively. Modules with low or no preservation were considered PP40-specific, reflecting sound-responsive gene networks. The royalblue module is highlighted with a blue arrow. (**C**) Module co-expression similarity between anterior and posterior brain tissues. For each module, the Pearson correlation between the upper-triangular elements of gene–gene correlation matrices derived from anterior and posterior brain samples was computed. Higher similarity values indicate conserved co-expression structures across regions, whereas lower values indicate regional divergence in network organization.

To uncover hub genes linked to the processing of attractive sounds, we applied WGCNA to transcriptomes of the anterior and posterior brain tissue, defining five traits: degree of attraction, PP10, PP80, PP40, and anterior brain. Among 22 identified modules, three modules (indicated by royalblue, grey60, and black) showed significant correlations with PP40 and/or behavioral preference (Figure 3A). Royalblue and grey60 modules were positively associated with the degree of attraction, PP40, and anterior brain, whereas black was correlated only with degree of attraction and PP40. In all three modules, gene significance (GS, the correlation between individual gene expression and the trait of interest) was significantly correlated with module membership (MM, the correlation between gene expression and the module eigengene) (Figure S2), indicating that genes most strongly associated with behavioral traits were also central within their respective co-expression networks.

We then assessed module preservation across acoustic conditions using the WGCNA modulePreservation() function (Figure 3B). The royalblue module exhibited low Zsummary scores (Zsummary = 2.26–3.86, Table S1) in comparisons between PP40 and NC, PP10, or PP80, indicating weak preservation of its co-expression structure under PP40 stimulation. Such low preservation suggests that the royalblue module undergoes substantial reorganization specifically in response to PP40, reflecting a PP40-specific transcriptional network. In contrast, most other modules, including grey60, showed moderate to high preservation (Zsummary > 10), indicating that their co-expression patterns remained largely conserved across acoustic conditions and were not specifically altered by PP40. The grey60 module therefore represents a relatively stable transcriptional program that was not specifically affected by PP40 stimulation, and was not pursued further in subsequent analyses.

Finally, we evaluated the specificity of module co-expression patterns by comparing the anterior and posterior brain tissue (Figure 3C). The royalblue module showed markedly lower co-expression similarity between the two regions, indicating strong anterior brain specificity, whereas the black module showed no significant difference (*p* = 0.243) and was excluded from further analysis. Together, these results identify the royalblue module as an anterior brain-enriched, PP40-responsive co-expression network.

### 2.4. Defining Core Regulatory Genes in the Hub Module

The royalblue module, which exhibited the strongest and most specific association with the attractive pulse pattern PP40 in the anterior brain, was further analyzed using three complementary approaches to identify potential molecular drivers.

First, hub gene identification was performed using cytoHubba with the MCC algorithm yielded a robust set of top consensuses hub genes. The top ranked hub genes were *GbOrb2* (Translational regulator orb2), *GbDnm* (Dynamin), *Gbgl* (Protein glass), *GbCadN* (Cadherin-related tumor suppressor), *GbNCadN* (Neural-cadherin), *Gbpum* (Pumilio 2), and *GbRbcn-3A* (Rabconnectin-3A) (Table 1).

**Figure 4:**
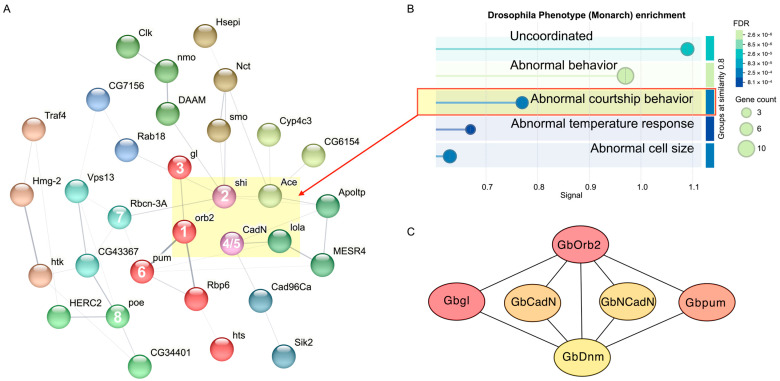
Protein–Protein Interaction Network Based on Genes from the royalblue Module. (**A**) Protein–Protein Interaction (PPI) network: The network illustrates the interactions among genes from the royalblue module. Each node represents a gene, and nodes are color-coded according to clusters identified by MCL clustering analysis. The network was constructed using *Drosophila* orthologs and visualized with STRING. The numbers in the nodes indicate the top-ranked nodes in the cricket network. (**B**) Drosophila Phenotype (Monarch) enrichment analysis: The plot shows the enrichment of different Drosophila phenotypes based on genes from the royalblue module. The *x*-axis represents the significance level (FDR), and the *y*-axis represents the signal value for each phenotype. The size of the circles reflects the number of genes associated with each phenotype. (**C**) Core regulatory network of the royalblue module: The PPI network of the six core nodes identified from the royalblue module. Edges represent interactions with weight > 0.25.

Second, we mapped the 56 protein sequences of these royalblue genes to *Drosophila* orthologs using STRING to construct a protein–protein interaction (PPI) network (Figure 4A). The resulting network comprised 38 connected *Drosophila* nodes, exhibited significant PPI enrichment (*p* = 4.22 × 10^−6^), and included key phenotypes related to abnormal behaviors, developmental lethality, and increased mortality (Table S2). Phenotype enrichment analysis identified several significantly enriched phenotypic categories, including “abnormal courtship behavior” (FBcv:0000399, false discovery rate = 0.0021, Figure 4B). Five *Drosophila* orthologs were enriched in this phenotype, including shi (Dynamin), orb2 (Translational regulator orb2), Ace (Acetylcholinesterase), CadN (Cadherin-related tumor suppressor), and lola (Longitudinals lacking protein).

Third, we performed unsupervised MCL clustering of the PPI network to identify tightly connected submodules. The largest cluster (Cluster 1) contained five genes, namely *orb2*, *glass*, *pum*, *Rbp6*, and *hts*. They were significantly enriched for GO terms related to translation regulation and messenger ribonucleoprotein complex. Notably, four of these genes (orb2, glass, pum and Rbp6) encode RNA-binding proteins known to regulate mRNA localization and translation in neurons, suggesting a potential post-transcriptional regulation of gene expression in response to the calling song.

To refine the core regulatory network, we focused on the top 6 genes identified through our network analyses, which were selected based on their centrality and importance in the PPI network. These genes, in order of significance, are *GbOrb2*, *Gbgl*, *Gbpum*, *GbDnm*, *GbCadN*, and *GbNCadN*. The first three genes (*orb2*, *gl*, and *pum*) clustered together in the *Drosophila* PPI network (Figure 4B), suggesting a tightly connected post-transcriptional regulatory network. The remaining three genes (*Dnm*, *CadNs*) were associated with the *Drosophila* abnormal courtship behavior phenotype, linking them to the context of auditory processing.

These six genes were also identified as the top 10 MCC consensus nodes in the royalblue module of the cricket network, based on the Maximal Clique Centrality (MCC) scores calculated by cytoHubba. The high MCC scores reflect their central roles in network connectivity, confirming their importance as key molecular drivers within the royalblue module. We then extracted these genes and their corresponding edges from the cytoscape input file generated by WGCNA, applying a weight threshold of >0.25 to filter significant interactions. The final core regulatory network reveals the key regulatory interactions which may be involved in forming the molecular response for integrating and processing attractive conspecific pulse patterns (Figure 4C).

## 3. Discussion

We investigated transcriptomic changes induced by acoustic stimulation which may support the molecular mechanisms underlying auditory processing of species-specific signals and phonotactic behavior in *G*. *bimaculatus*. Our behavioral tests using a T-maze free choice paradigm demonstrated that female crickets strongly preferred the calling song stimulus (PP40) over no-sound control and any other condition, with no significant preference for the PP10, PP80, or silent control conditions. This stereotyped behavioral response pattern reflects the species-typical auditory preference as reported before [5].

Unlike previous studies [5] where PP10 and PP80 elicited weak but measurable phonotactic responses, our experiments showed no distinct response for these stimuli. This difference likely reflects methodological distinctions. The consistent behavioral response across individuals established a clear framework for linking gene expression to auditory-driven behavior. We grouped females by stimulus condition (PP40, PP10, PP80, or no-sound control) and compared their transcriptional profiles to identify molecular signatures underlying exposure to species-specific sounds versus non-responsiveness.

Transcriptomic profiling across brain regions revealed that PP40-induced gene expression changes were predominantly localized to the anterior brain. The number of downregulated genes was greater than that of upregulated genes, suggesting that certain biological processes may be suppressed to allocate resources toward auditory processing. Neurophysiological studies have demonstrated that the anterior brain, particularly the lateral protocerebrum, houses the neural circuits responsible for auditory pattern recognition and phonotactic decision-making [3,14]. To identify functionally coordinated gene networks activated by species-specific acoustic stimuli, we applied WGCNA, which revealed a distinct gene module (royalblue) that was significantly correlated with the PP40 stimulus and the anterior brain. Phenotype enrichment and protein–protein interaction analyses further supported the functional relevance of this module to mating behavior. In *Drosophila* phenotype enrichment based on *Drosophila* orthologs revealed that genes from the royalblue module were significantly associated with the term “abnormal *Drosophila* courtship behavior” (FBcv:0000399). This phenotype encompasses multiple *Drosophila* courtship defects, including abnormalities in male song production and altered courtship behavioral rates. Phenotype enrichment analysis of the *Drosophila* orthologs corresponding to genes in the royalblue module revealed significant association with courtship behavior, a finding that reinforces the functional relevance of this gene network. *Drosophila* courtship and cricket phonotaxis are distinct behaviors, yet both use acoustic signals as key cues for mate-related interactions, suggesting the involvement of partially similar functional molecular pathways underlying acoustic communication. Notably, the royalblue module in crickets was specifically associated with responses to the calling song (PP40), which is a signal central to mate recognition. This may indicate a cross-species functional similarity and suggest that genes within this module may participate in molecular processes relevant to auditory processing and reproductive decision-making in insects.

Network analysis identified six highly connected hub genes within the calling song-responsive module: *GbOrb2*, *Gbgl*, *Gbpum*, *GbDnm*, *GbCadN*, and *GbNCadN*. Based on their central positions in the co-expression network and known functions in other systems, these genes are inferred to potentially contribute to auditory processing related to mate recognition in crickets. A prominent candidate is *GbOrb2*, a central node in the co-expression network. In *Drosophila*, *Orb2* encodes a CPEB-type RNA-binding protein required for long-term memory [15,16], including memory formation during courtship conditioning, a behavioral paradigm in which males learn from unsuccessful mating attempts and subsequently reduce courtship toward non-receptive females [17]. *Orb2* functions as a molecular switch regulated by its oligomerization state [18,19,20]. In its monomeric form, *Orb2* represses translation by reducing poly(A) tail length, while neuronal stimulation triggers stable amyloid-like oligomers that instead activate translation [19,21]. This oligomerization occurs within minutes of stimulation in *Drosophila* neurons [20], a timescale that could hypothetically correspond to the rapid behavioral responses observed in cricket phonotaxis.

Given its central role in the co-expression network and the functional insights from *Drosophila* studies, we propose a working network-based hypothesis that *GbOrb2* may function as an activity-dependent translational regulator that facilitates the processing of species-specific acoustic patterns, though this hypothesis requires direct experimental validation. The rhythmic nature of cricket calling songs, consisting of discrete chirps separated by silent intervals, may be particularly well-suited to activity-dependent regulatory mechanisms like *Orb2*-mediated translational control. One speculative scenario, prompted by the network model, is that repeated exposure to the temporal pattern of PP40 could trigger *GbOrb2* oligomerization in anterior brain regions, potentially facilitating the translation of downstream targets involved in acute auditory processing or in subsequent memory formation, as observed in flies [15,17]. However, several critical questions remain unanswered: whether *GbOrb2* indeed undergoes oligomerization in response to acoustic stimulation, which specific mRNAs it regulates in the cricket brain, and whether this regulation occurs on timescales relevant to behavioral choice (seconds to minutes) or longer-term memory consolidation (hours to days).

The other hub genes in the calling song-responsive module complement these post-transcriptional mechanisms with additional regulatory layers. *Gbgl*, homologous to the *Drosophila* transcription factor *glass*, may provide transcriptional control. However, whether it functions in auditory system development or is reactivated in adult neural plasticity remains unclear [22,23]. *Gbpum*, a Pumilio-family RNA-binding protein, likely contributes additional post-transcriptional regulation [24,25]. *GbDnm* (dynamin) is essential for synaptic vesicle recycling and neurotransmission [26]. Dynamin also functions in synaptic plasticity and learning in mushroom body neurons [27], where it contributes to dendritic pruning and synaptic refinement [28]. *GbCadNs* were homologous to *Drosophila N-cadherin*, mediating homophilic neuronal interactions and neural circuit assembly through layer-specific axon targeting and synaptic organization [29,30].

It’s important to note that the functional roles of these genes have been primarily characterized in the context of *Drosophila* courtship behavior, which differs from cricket calling song communication. Moreover, the acute nature of our experimental paradigm (1 min acoustic exposure) differs from the timescales over which *Orb2* function has been characterized in *Drosophila*, which typically involve long-term memory persisting hours to days post-training. *Drosophila* courtship song is a close-range acoustic signal produced during male-female interactions that enhances female receptivity and facilitates mating decisions [31,32]. In contrast, cricket calling songs are long-range signals that attract distant conspecific females and initiate phonotactic approach behavior. Males then switch to courtship song once females are at close range [33], representing distinct stages in mate acquisition. Despite these ethological differences, both cricket calling song recognition and *Drosophila* courtship song perception require discrimination of species-specific temporal patterns and may involve formation of acoustic representations or memories. The conservation of genes like *Orb2*, *Pumilio*, and others across these contexts suggests that certain molecular mechanisms, particularly activity-dependent post-transcriptional regulation and synaptic modification, may be fundamental to acoustic pattern processing regardless of the species-specific behavioral context. However, whether these genes play analogous roles in the distinct neural circuits and timescales associated with long-range attraction versus close-range courtship evaluation remains an open question for comparative study. We emphasize that *GbOrb2* and other hub genes identified in this study represent candidates for future functional investigation rather than validated mediators of phonotactic behavior. In addition, the acute nature of the acoustic stimulation paradigm represents a limitation of this study. Although we detected robust transcriptomic differences following 1 min of acoustic exposure, the lack of multiple stimulation durations prevents us from characterizing the temporal dynamics of gene expression responses. Future studies incorporating graded stimulation lengths and post-stimulus sampling at multiple time points will be important for distinguishing immediate transcriptional responses from later, potentially plasticity-related changes.

Interestingly, we did not observe significant differential expression of *fruitless* (*fru*) genes in response to calling song stimulation (all FDR > 0.4; Table S3). In *Drosophila*, fruitless encodes a transcription factor that plays a master regulatory role in establishing sexually dimorphic neural circuits underlying courtship behavior [34,35]. However, unlike the situation in *Drosophila* and other holometabolous insects, the *Gryllus fruitless* homolog does not produce sex-specific protein isoforms and is not integrated into the neural sex-determination system [36]. In *Gryllus bimaculatus*, *fruitless* may instead work together with *doublesex* (*dsx*) to form the neural framework necessary for sex-specific behaviors during development [37], rather than through acute activity-dependent regulation. The absence of calling song-induced fruitless expression changes is consistent with this developmental rather than stimulus-responsive role. One interpretation is that calling song recognition may operate within neural circuits that are established during development and sexually differentiated by *fruitless*, with the acute response to acoustic stimulation potentially mediated through post-transcriptional and synaptic regulatory mechanisms (*GbOrb2*, *Gbpum*, *GbDnm*, *GbCadN*) rather than through restructuring of circuit architecture.

Together, the down and up-regulated genes suggest that calling song exposure triggers comprehensive multi-level molecular regulation spanning transcriptional control (*Gbgl*), post-transcriptional modulation (*GbOrb2*, *Gbpum*), synaptic function (*GbDnm*), and neural connectivity establishment (*GbCadNs*), indicating a coordinated gene regulatory framework for the species-specific acoustic signal processing circuit.

In conclusion, our transcriptomic analysis has revealed a complex gene regulatory network activated by cricket calling song exposure, encompassing multiple layers of cellular control from transcriptional programming to synaptic dynamics. The identification of the similar regulatory mechanisms in the cricket and fruit fly suggests that acoustic communication systems may share some molecular principles across arthropod species, despite their diverse evolutionary origins and behavioral contexts. The activity-dependent nature of several key regulators, particularly *GbOrb2′s* oligomerization switch, highlights the importance of stimulus-responsive mechanisms in processing intermittent calling song signals. Future functional studies of these candidate genes will be essential to validate their specific roles in calling song recognition and to elucidate how this regulatory network integrates and is linked to neural processing that underlies the behavioral responses critical for phonotactic behavior in crickets.

## 4. Materials and Methods

### 4.1. Animals

All experiments used intact adult female cricket (*G*. *bimaculatus*), at 10–20 days post-ecdysis. Last-instar nymphs were separated from the colony at Xianghu Laboratory, Hangzhou, China. Selected females were inspected to ensure the integrity of their tympanic membranes and auditory spiracles. The colony was maintained at 26–28 °C and 60% relative humidity under a 16:8 h light:dark cycle. Animals were fed *ad libitum* with potatoes, freeze-dried fish, cereals and water.

### 4.2. Acoustic Stimuli

Based on previous phonotaxis experiments [38], we used three pulse patterns which elicit differential phonotactic and neural responses [5,39]. These were a species-specific calling song analogue, PP40 (40 ms pulse period), and two less attractive chirps, PP10 (10 ms pulse period) and PP80 (80 ms pulse period (Figure 1A). All chirps consisted of four pulses, composed of equal pulse and interval durations (e.g., PP40 comprised 20 ms pulses separated by 20 ms intervals). This resulted in chirp durations of 35 ms, 140 ms, and 280 ms for PP10, PP40, and PP80, respectively. Sound stimuli were generated using Cool Edit Pro (Version 2.1, Syntrillium Software, Phoenix, AZ, USA) and delivered through HP S01 Bluetooth speakers (HP Inc., Palo Alto, CA, USA). The carrier frequency of all stimuli was 4.8 kHz, and the sound pressure level (SPL) was calibrated to 75 dB SPL using a sound level meter (Aihua Instruments Co., Hangzhou, China).

### 4.3. Two-Choice Behavioral Assay

Phonotaxis behavior was assessed using a custom-designed, 3D-printed T-maze (610 mm in total length), featuring a central vertical tube for releasing the insects (Figure 1B). The T-maze paradigm was used to establish categorical behavioral preferences for different pulse patterns under naturalistic conditions, providing the behavioral foundation for selecting stimulus conditions in the parallel transcriptomic experiment. The walls of the maze consisted of a fine mesh to minimize acoustic interference. All bioassays were conducted within a soundproof chamber under dark conditions at 24–26 °C and 60% relative humidity.

Two speakers were positioned 30 mm from the opposite ends of the T-maze’s horizontal arms. For each trial, one speaker played an acoustic stimulus while the other end served as a no-sound control (Table 1). The stimulus side was alternated between trials. Chirps were repeated with an inter-chirp interval equal to the chirp duration until the cricket reached one of the T-maze exits (defined as when any body part crossed the exit threshold). The sound pressure level for the stimuli was calibrated to 75 dB SPL at the central release point of the T-maze.

Individual crickets were released from the central vertical tube, and one acoustic stimulus was played immediately upon the release. A choice was recorded once the cricket passed one of the horizontal arms of the T-maze and exited through the opening. Each cricket was exposed to only one sound stimulus during a single trial, and only one choice was recorded per individual. Individuals that failed to make a choice within 5 min were classified as non-responsive. For each acoustic condition, five trials with 10 individuals per trial were tested (N = 5, *n* = 50). These experiments were used to establish the likelihood of phonotactic active females in the colony.

### 4.4. RNA-Seq Sampling and Bioinformatic Analyses

To identify transcriptional responses associated with auditory processing of conspecific calling songs, we performed RNA-seq on brain tissues from female crickets exposed to different acoustic stimuli. This experiment was conducted in parallel with, but independently from, the T-maze phonotaxis assays described above. Each cricket was exposed to only one acoustic condition and immediately dissected for sample collection. Female crickets were individually secured ventral-side down on a wax plate using insect pins to gently hold the body without piercing the cuticle, preventing movement during acoustic exposure. Each cricket was then exposed to a single acoustic stimulus (PP10, PP40, PP80, or silence) for 1 min in a sound-attenuated chamber. After the acoustic stimulus ended, the animal was immediately removed from the chamber, placed on ice, and dissected; the head was severed and transferred to an ice-cold glass slide within approximately 5 min of stimulus termination.

Under a stereomicroscope, the head capsule was opened by making a longitudinal incision along the dorsal midline and carefully removing the dorsal cuticle to expose the brain. The brain was extracted using fine forceps, severing all nerve connections, connectives and associated tissues. All dissection procedures were performed on ice to minimize RNA degradation. Following extraction, each brain was immediately subdivided into an anterior and posterior region. An incision was made perpendicular to the anterior–posterior axis (Figure 2A). The anterior region primarily contained the protocerebrum, including the mushroom bodies and protocerebral bridge, while the posterior region contained the central complex, antennal lobes, and associated neuropils.

Three biological replicates were prepared per condition, with each replicate consisting of pooled tissues from three individuals. Anterior brain and posterior brain samples were pooled separately, yielding 24 total samples (4 conditions × 2 tissue types × 3 biological replicates). Samples were immediately frozen in liquid nitrogen and stored at −80 °C.

Total RNA was extracted from the 24 pooled samples using Trizol reagent (Takara Bio Inc., Kusatsu, Japan, cat#: 9109). RNA concentration and purity were measured using a NanoDrop spectrophotometer (Thermo Fisher Scientific, Waltham, MA, USA). RNA samples with A260/A280 ratios between 1.8–2.0 and A260/A230 ratios > 1.8 were considered acceptable. RNA integrity was assessed using an Agilent 2100 Bioanalyzer (Agilent Technologies, Santa Clara, CA, USA), samples with RNA Integrity Number (RIN) ≥ 8.0 and concentrations ≥ 1 µg were used for library construction.

mRNA was enriched using oligo (dT) magnetic beads and fragmented in NEB Fragmentation Buffer. Strand-specific RNA-seq libraries were prepared using the NEBNext Ultra II Directional RNA Library Prep Kit (New England Biolabs, Ipswich, MA, USA, cat#: E7760S) following the manufacturer’s protocol. Library quality was assessed using Qubit 2.0 Fluorometer (Thermo Fisher Scientific) for initial quantification and Agilent 2100 Bioanalyzer for insert size determination. Libraries with effective concentrations > 2 nM were pooled and sequenced on the Illumina NovaSeq X Plus platform (Illumina, San Diego, CA, USA) with paired-end 150 bp reads. Raw RNA-seq data have been deposited in the China National Center for Bioinformation (CNCB) under the submission number: PRJCA050865.

Raw sequencing reads were processed using fastp (version 0.23.1) [40], for quality control, including adapter trimming and removal of low-quality reads. Clean reads were aligned to the *G. bimaculatus* reference genome (GenBank assembly: GCA_017312745.1) using HISAT2 (version 2.2.1) [41]. Alignment quality was assessed using QualiMap (version 2.2.2-dev) [42]. Transcript assembly and quantification were performed using StringTie (version 2.1.5) [43]. Novel transcripts were compared with reference annotations using gffcompare (version 0.12.6) [44]. Gene expression levels were quantified as read counts using featureCounts (version 2.0.1) [45]. Differential expression analysis was performed using DESeq2 (version 1.26.0) [46] in R (version 4.3.2). Differentially expressed genes (DEGs) were identified based on an adjusted *p*-value (FDR) < 0.05 and an absolute log_2_(fold change) > 1. Gene Ontology (GO) [47,48] and Kyoto Encyclopedia of Genes and Genomes (KEGG) [49,50] pathway enrichment analyses were performed using clusterProfiler (version 3.14.3) [51]. GO terms and KEGG pathways with *q*-value < 0.05 were considered significantly enriched.

### 4.5. Weighted Gene Co-Expression Network Analysis (WGCNA)

To identify groups of functionally related genes that share similar expression patterns in response to different acoustic pulse pattern, we performed Weighted Gene Co-expression Network Analysis (WGCNA) to the transcriptomes of anterior and posterior brain samples [52].

Gene expression levels were normalized as transcripts per million (TPM) using StringTie (version 2.1.5). Co-expression network construction and module detection were performed using the WGCNA package (version 1.72-1) in R. A signed hybrid network was constructed with a soft-thresholding power of 3, selected based on scale-free topology criteria (Figure S3). Modules were identified using hierarchical clustering with a minimum module size of 30 genes and a merge cut height of 0.25. Each co-expression module was assigned a unique color label (e.g., “royalblue”, “turquoise”) by WGCNA for visualization purposes, while genes that could not be classified into any other module were assigned to a grey module. These color labels are automatically generated and do not have intrinsic biological meaning. Module-trait correlations were calculated using a trait matrix with five variables: (1) stimuli-specific traits (PP10, PP40, PP80; binary; 1 = exposed, 0 = not exposed), (2) degree of attraction (quantitative: PP40 = 1, PP10/PP80 = 0.1, silence = 0; based on behavioral data from [5], and (3) brain region (binary: anterior = 1, posterior = 0). This approach enabled identification of modules associated with specific acoustic patterns and brain regions.

Candidate modules were identified using a three-criteria filtering approach. First, we assessed module preservation across acoustic conditions using the modulePreservation() function in WGCNA [53]. Module preservation statistics were computed between PP40 and each of the other three treatments (NC, PP10, and PP80) using permutation-based Zsummary statistics (200 permutations). Thresholds were applied as per [53], with Zsummary < 2 indicating non-preserved modules, 2–10 indicating weak to moderate preservation, and >10 representing strongly preserved modules. Modules showing low or no preservation (Zsummary < 10) were considered to have undergone specific reorganization under PP40 stimulation, and were therefore treated as candidate networks potentially associated with PP40-specific transcriptional responses.

Second, to examine brain region specificity, we quantified the similarity of co-expression patterns between the anterior and posterior brain regions. For each module, we computed the Pearson correlation matrices among member genes within anterior and posterior samples separately, and then calculated the Pearson correlation between the upper-triangular elements of these matrices. This similarity score ranges from −1 to 1, where values approaching 1 indicate highly similar co-expression patterns between brain regions, and values near 0 indicate substantial divergence, and negative values indicate opposite correlation patterns. Third, we assessed the relationship between Gene Significance (GS) and Module Membership (MM) for each module. Modules with a significant correlation (*p* < 0.05) between GS and MM were considered biologically meaningful and retained for further functional and network analyses.

### 4.6. Hub Gene Identification and Functional Analysis

Genes from the selected candidate modules were used to construct a protein–protein interaction (PPI) network, which represents the physical or functional associations among proteins encoded by these genes. The PPI network allows identification of central regulatory nodes that may play key roles in the module’s biological functions. The protein sequences encoded by genes from the selected candidate modules were input into Cytoscape (version 3.10.3) [54] for visualization and topological analysis. To ensure robustness, we run the CytoHubba plugin 10 times and perform statistical analysis to identify hub genes [55]. Twelve centrality measures were calculated to identify key nodes and their roles in network connectivity. Top-ranking nodes were identified based on the MCC (Maximal Clique Centrality) scores. The top 10 genes that consistently ranking high across 10 runs were designated as hub gens.

Subsequently, the nodes from the hub module were input into the STRING database (version 12.0) [56], and generated a network based on homologous proteins in *Drosophila melanogaster*. A confidence threshold (minimum interaction score = 0.15) was applied to retain potential functional associations among homologous proteins. This threshold was chosen because the network was constructed from *Drosophila* homologs of cricket genes, and using a higher cutoff would likely exclude biologically relevant but less well-characterized interactions due to interspecies divergence. Functional networks based on these homologous proteins were then constructed and subjected to clustering and enrichment analyses to explore the functional relevance of these genes activated by cricket auditory processing. Phenotype enrichment analysis was performed using the integrated *Drosophila* Monarch function within the STRING platform, which queries phenotype-genotype associations from the Monarch Initiative database [57].

## Figures and Tables

**Table 1:** Top10 MCC consensus nodes in royalblue module.

ID	Annotation	String Node	Avg_Rank	Min_Rank	Rank_Std	Avg_Score
GBIM_04859	Translational regulator orb2	orb2	2.8	1	2.35	39.2
GBIM_20807	Dynamin	shi	7.3	1	9.36	31.7
GBIM_07540	Protein glass	gl	8	1	8.03	29.8
GBIM_06818	Cadherin-related tumor suppressor	CadN	9	2	5.62	22.2
GBIM_06817	Neural-cadherin	CadN	11.2	3	9.04	23.4
GBIM_06309	Pumilio 2	pum	13.4	6	8.13	18.2
GBIM_14432	Rabconnectin-3A	Rbcn-3A	14.8	2	11.36	23.2
GBIM_20222	Protein purity of essence	poe	15.1	2	9.36	19.1
GBIM_18457	Protein of unknown function		15.5	1	10.22	17.4
GBIM_12519	E3 ubiquitin-protein ligase Ufd4	Ufd4	15.6	4	9.13	17.7

Note: Results from consensus analysis of MCC (Maximal Clique Centrality) algorithm across 10 independent runs. avg_rank: average ranking (lower = more important); rank_std: ranking standard deviation (lower = more stable); min_rank ranking range; avg_score: average MCC score (higher = more important).

## Data Availability

The raw RNA-seq data presented in this study are openly available in the China National Center for Bioinformation (CNCB) at https://ngdc.cncb.ac.cn/gsa/ (accessed on 3 August 2025), reference number PRJCA050865. Other data supporting the findings of this study are available from the corresponding author upon reasonable request.

## Supplementary Materials

The following supporting information can be downloaded at: https://www.mdpi.com/article/10.3390/ijms27020706/s1.

## Abbreviations

The following abbreviations are used in this manuscript:

Q	anterior brain
H	posterior brain
NC	no sound control
PP10	pulse period 10 ms
PP40	pulse period 40 ms
PP80	pulse period 80 ms
