# Supplementary material for: A Co-Expressed Cluster of Genes in the Anterior Brain of Female Crickets Activated by a Species-Specific Calling Song
Source: Int J Mol Sci. 2026 Jan 10;27(2):706. doi: 10.3390/ijms27020706 (PMC12841516; doi:10.3390/ijms27020706)
Supplement: Supplementary file 1 [file ijms-27-00706-s001.zip › ijms-4031713-supplementary.pdf]

Table S1. Module preservation statistics across acoustic stimulations

Reference	Test	Module	Zsummary	Zdensity	Zconnectivity	medianRank	moduleSize
**PP40**	NC	black	-1.172828043	-3.355243846	1.00958776	21	385
**PP40**	NC	blue	22.74226035	6.514490381	38.97003032	9	1000
**PP40**	NC	brown	18.14907612	2.74057249	33.55757974	10	889
**PP40**	NC	cyan	5.568473146	4.316730287	6.820216004	14	117
**PP40**	NC	darkgreen	-0.181482295	-1.315320881	0.95235629	21	49
**PP40**	NC	darkred	-0.315716626	-1.484920498	0.853487247	20	52
**PP40**	NC	gold	28.55656528	11.13525479	45.97787577	6	1000
**PP40**	NC	green	12.73072025	1.509133802	23.95230671	16	502
**PP40**	NC	greenyellow	7.580798797	2.623516835	12.53808076	10	149
**PP40**	NC	grey	-2.830169557	-5.835618168	0.175279054	24	536
**PP40**	NC	grey60	9.087841876	5.406025292	12.76965846	3	97
**PP40**	NC	lightcyan	2.507621897	-0.588114226	5.603358021	17	99
**PP40**	NC	lightgreen	6.029384539	2.050879892	10.00788919	6	94
**PP40**	NC	lightyellow	3.996837611	2.864562158	5.129113063	5	76
**PP40**	NC	magenta	6.28630336	-0.042181609	12.61478833	18	170
**PP40**	NC	midnightblue	4.420147116	0.475620497	8.364673735	11	99
**PP40**	NC	pink	10.77529743	6.753561833	14.79703303	4	178
**PP40**	NC	purple	6.223652916	0.573308998	11.87399684	13	170

**PP40**	NC	red	5.354417656	-0.601521799	11.31035711	18	479
**PP40**	NC	royalblue	2.942304316	3.203519857	2.681088775	18	56
**PP40**	NC	salmon	-1.070516296	-2.976750795	0.835718202	23	133
**PP40**	NC	tan	6.656207956	3.059356513	10.2530594	9	144
**PP40**	NC	turquoise	35.1418737	25.20500112	45.07874629	1	1000
**PP40**	NC	yellow	19.47813085	9.110691121	29.84557057	7	658
**PP40**	PP10	black	0.60447381	-2.405616978	3.614564599	22	385
**PP40**	PP10	blue	23.85105844	14.24826684	33.45385004	9	1000
**PP40**	PP10	brown	27.92571464	19.65124031	36.20018896	8	889
**PP40**	PP10	cyan	2.766931039	-0.622119164	6.155981242	20	117
**PP40**	PP10	darkgreen	3.878400935	1.681009905	6.075791965	15	49
**PP40**	PP10	darkred	0.351941244	-0.753836372	1.45771886	20	52
**PP40**	PP10	gold	24.03330646	9.644490581	38.42212233	11	1000
**PP40**	PP10	green	9.712111102	-0.234523791	19.658746	17	502
**PP40**	PP10	greenyellow	11.3118885	5.914821962	16.70895503	11	149
**PP40**	PP10	grey	-1.614753192	-5.572025954	2.34251957	24	536
**PP40**	PP10	grey60	10.20182453	7.811834875	12.59181419	5	97
**PP40**	PP10	lightcyan	6.924006257	5.705354339	8.142658176	8	99
**PP40**	PP10	lightgreen	11.19515764	14.55519561	7.835119679	4	94
**PP40**	PP10	lightyellow	9.457306013	8.313768991	10.60084304	2	76

**PP40**	PP10	magenta	10.45566484	4.079607191	16.83172248	12	170
**PP40**	PP10	midnightblue	10.87222394	8.05820169	13.68624619	3	99
**PP40**	PP10	pink	10.97063897	7.905008445	14.03626949	7	178
**PP40**	PP10	purple	8.53707026	2.695184542	14.37895598	15	170
**PP40**	PP10	red	2.5582337	-1.124525214	6.240992613	19	479
**PP40**	PP10	royalblue	2.255083451	0.939523978	3.570642924	17	56
**PP40**	PP10	salmon	3.611606392	6.148555115	1.074657669	22	133
**PP40**	PP10	tan	6.219326756	2.715691922	9.722961589	16	144
**PP40**	PP10	turquoise	35.41934563	28.3801052	42.45858607	3	1000
**PP40**	PP10	yellow	22.8679067	10.91915358	34.81665982	10	658
**PP40**	PP80	black	-0.526624725	-2.26636882	1.213119369	21	385
**PP40**	PP80	blue	19.03624386	5.137333911	32.9351538	11	1000
**PP40**	PP80	brown	16.34756517	0.35882927	32.33630106	16	889
**PP40**	PP80	cyan	0.804432123	-1.925318514	3.53418276	21	117
**PP40**	PP80	darkgreen	6.091299046	3.983126598	8.199471494	10	49
**PP40**	PP80	darkred	4.709068518	5.240110543	4.178026493	8	52
**PP40**	PP80	gold	28.06940483	10.12085437	46.01795529	5	1000
**PP40**	PP80	green	17.80258003	10.64444811	24.96071196	6	502
**PP40**	PP80	greenyellow	13.81969676	8.799511792	18.83988173	3	149
**PP40**	PP80	grey	-2.861652052	-6.084623401	0.361319298	24	536

**PP40**	PP80	grey60	9.500779695	5.8787429	13.12281649	5	97
**PP40**	PP80	lightcyan	-0.876484309	-2.729132351	0.976163733	21	99
**PP40**	PP80	lightgreen	4.525687157	0.929407199	8.121967115	10	94
**PP40**	PP80	lightyellow	6.513671252	3.300286009	9.727056495	3	76
**PP40**	PP80	magenta	7.771732915	0.477394377	15.06607145	14	170
**PP40**	PP80	midnightblue	2.251047059	-0.688722968	5.190817086	16	99
**PP40**	PP80	pink	9.668914751	2.934070506	16.403759	6	178
**PP40**	PP80	purple	6.397126108	0.455187383	12.33906483	15	170
**PP40**	PP80	red	14.34763816	18.25399817	10.44127814	16	479
**PP40**	PP80	royalblue	3.856541761	4.822164953	2.890918569	18	56
**PP40**	PP80	salmon	-1.482375431	-3.068292368	0.103541505	23	133
**PP40**	PP80	tan	2.200476587	0.664314932	3.736638242	15	144
**PP40**	PP80	turquoise	35.74223903	26.55433826	44.9301398	2	1000
**PP40**	PP80	yellow	16.62862237	2.465282116	30.79196262	14	658

Table S2. Key Phenotypes in the *Drosophila* PPI Network

Phenotype	Discription	Count In Network	Strength	Fdr	Signal
FBcv:0000416	Uncoordinated	5 of 133	1.16	0.0022	0.8
FBcv:0000399	Abnormal courtship behavior	5 of 152	1.11	0.0022	0.78
FBcv:0000387	Abnormal behavior	8 of 424	0.86	0.0018	0.67
FBcv:0002030	Lethal - all die during pupal stage	3 of 90	1.11	0.0167	0.57
FBcv:0000388	Abnormal gravitaxis	2 of 37	1.32	0.0304	0.53
FBcv:0000430	Abnormal cell polarity	3 of 114	1.01	0.0244	0.5
FBcv:0000428	Abnormal cell size	5 of 306	0.8	0.0167	0.48
FBcv:0000424	Abnormal cell death	7 of 582	0.67	0.0122	0.45
FBcv:0000392	Increased rate of movement	2 of 55	1.15	0.0479	0.45
FBcv:0000359	Decreased cell number	6 of 522	0.65	0.0185	0.42
FBcv:0000425	Increased cell death	6 of 554	0.62	0.0225	0.39
FBcv:0000375	Semi-fertile	3 of 171	0.83	0.0494	0.39
FBcv:0000351	Lethal	23 of 4340	0.31	0.0022	0.34
FBcv:0002001	Lethal - all die before end of first instar larval stage	9 of 1185	0.47	0.0223	0.34
FBcv:0000709	Abnormal cell number	7 of 826	0.52	0.0307	0.34
FBcv:0000435	Abnormal neuroanatomy	12 of 1836	0.4	0.0167	0.33
FBcv:0002005	Lethal - all die during P-stage	7 of 857	0.5	0.0347	0.33
FBcv:0002002	Lethal - all die before end of larval stage	13 of 2021	0.4	0.0167	0.32
FBcv:0002033	Lethal - all die during embryonic stage	8 of 1080	0.46	0.0346	0.31
FBcv:0002000	Lethal - all die before end of P-stage	17 of 3102	0.33	0.0139	0.3
FBcv:0002027	Lethal - all die before end of pupal stage	15 of 2600	0.35	0.0167	0.3
FBcv:0002015	Partially lethal	16 of 2917	0.33	0.0167	0.3
FBcv:0000385	Short lived	8 of 1170	0.42	0.0467	0.29
FBcv:0002019	Increased mortality during development	24 of 5330	0.24	0.0109	0.27
FBcv:0000352	Partially lethal - majority die	12 of 2142	0.34	0.0347	0.27

Table S3. Log2 Fold Changes and False Discovery Rates of *fruitless* Gene Expression in Cricket Across Multiple Comparisons

Gene ID	Description	HNC-vs-HCS	FDR	HNC-vs-HPP10	FDR	HNC-vs-HPP80	FDR	QNC-vs-QCS	FDR	QNC-vs-QPP10	FDR	QNC-vs-QPP80	FDR
GBIM_08079	Sex determination protein fruitless	-4.3219	0.98834612	-4.3219	0.99996029	-4.3219	0.98149265	-1.2224	0.96494332	-0.9005	0.99171827	-1	0.98364639
GBIM_08529	Sex determination protein fruitless	0.0408	0.98834612	-0.1103	0.99996029	-0.07	0.98733236	0.0682	0.9685297	0.0943	0.99171827	0.0655	0.98364639
GBIM_14708	Fruitless B isoform	-0.1711	0.84329694	-0.0323	0.99996029	-0.19	0.93617481	0.2323	0.50015972	0.2903	0.50044527	-0.0363	0.98364639
GBIM_14709	Fruitless H1 isoform	-0.1641	0.98834612	-0.1492	0.99996029	-0.5277	0.84641482	0.0527	0.98650472	0.0703	0.99171827	0.2023	0.9704807
GBIM_14710	Sex determination protein fruitless	-0.1365	0.89790798	-0.1127	0.99996029	-0.3761	0.44141236	0.1394	0.81398055	0.1989	0.86005202	0.0119	0.99989661

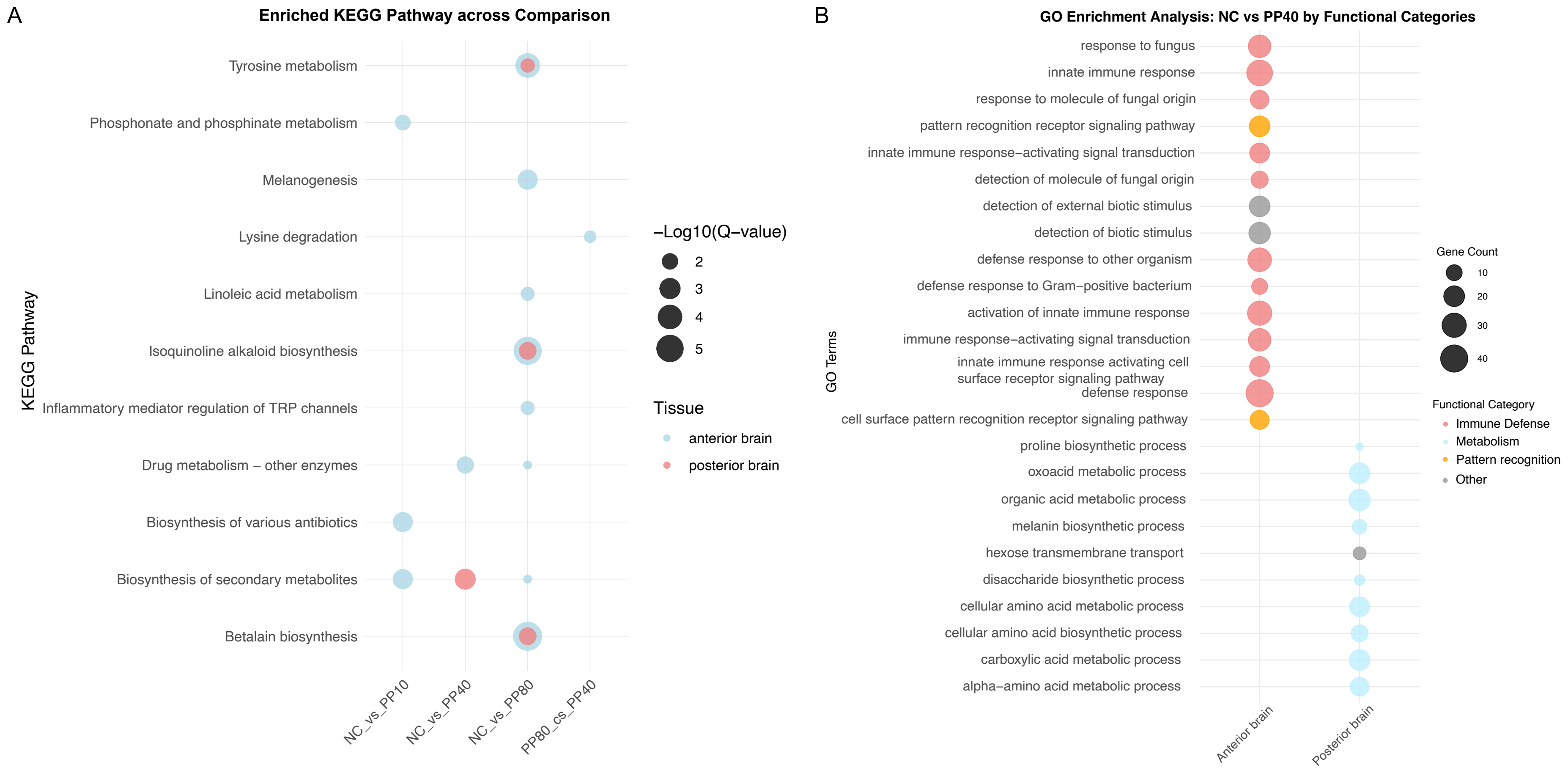

Figure S1. Region-Specific Molecular Responses to PP40 Stimulation. (A) KEGG pathway enrichment analysis in anterior and posterior brain regions; (B) GO enrichment analysis showing immune and metabolic processes.

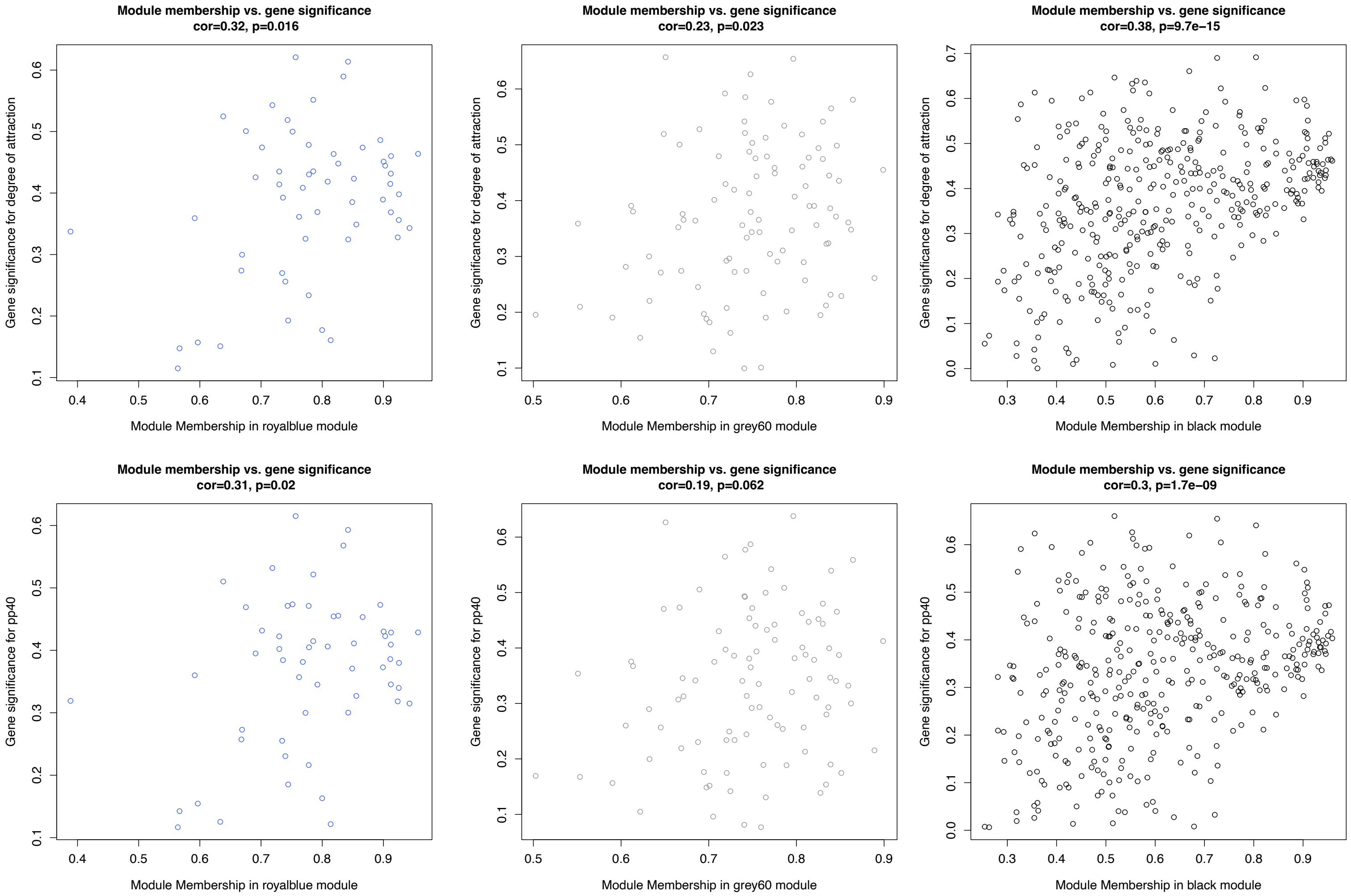

Figure S2. Gene Significance Correlates with Module Membership Across Three Co-Expression Modules.

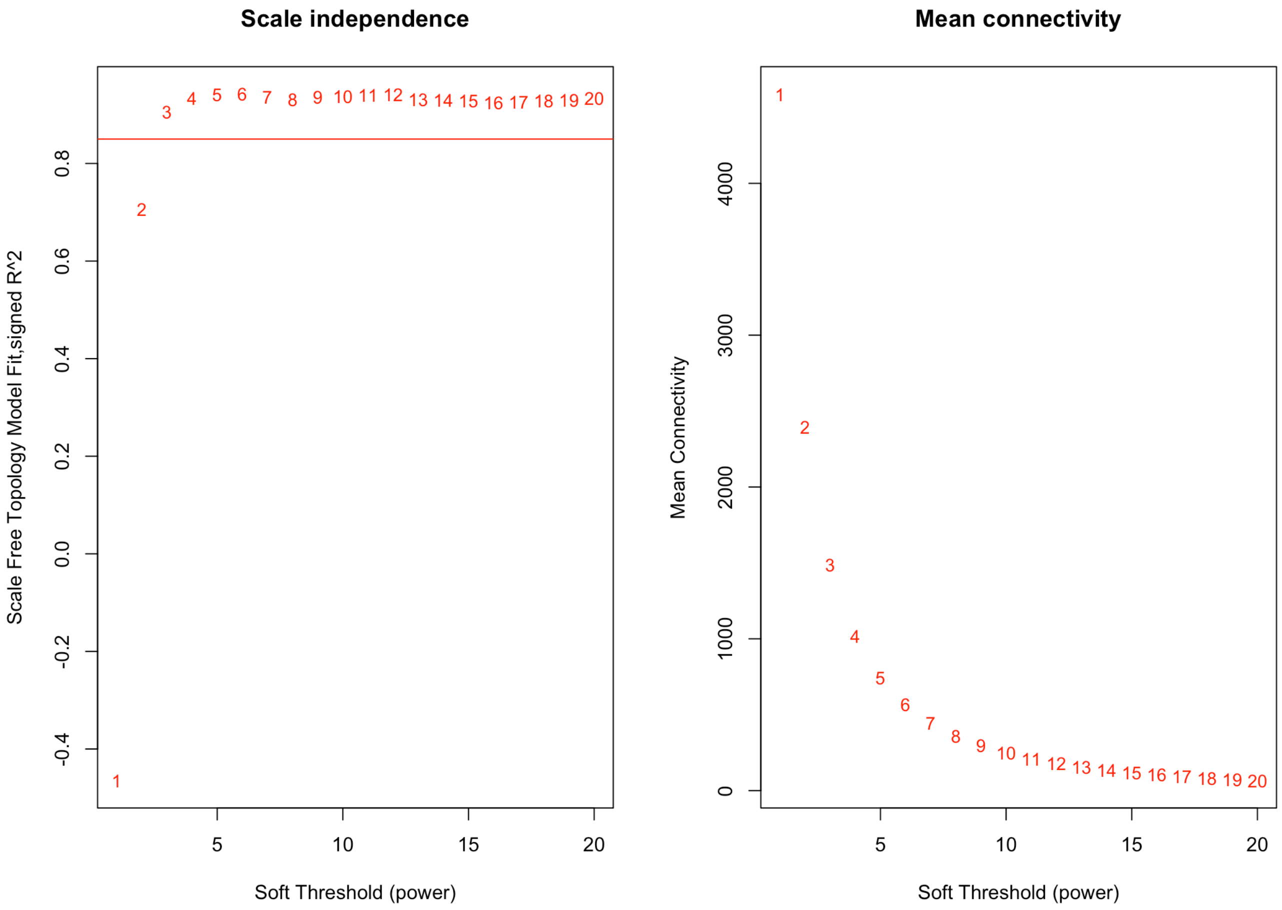

Figure S3. Determination of soft-thresholding power for WGCNA. (A) Scale-free topology model fit (R^2) as a function of soft-thresholding power. The red line indicates the threshold of $R^2 = 0.85$. Numbers indicate the corresponding soft-thresholding power. (B) Mean connectivity as a function of soft-thresholding power.
